# Amperometric Determination of Glucose at Physiological pH by an Electrode Modified with a Composite Ni/Al-Layered Double Hydroxide and Electrochemically Reduced Graphene Oxide

**DOI:** 10.3390/nano15151172

**Published:** 2025-07-30

**Authors:** Domenica Tonelli

**Affiliations:** Department of Industrial Chemistry “Toso Montanari”, University of Bologna, Via Piero Gobetti 85, 40129 Bologna, Italy; domenica.tonelli@unibo.it

**Keywords:** glucose detection, electrocatalysis, physiological pH, Ni/Al-layered double hydroxide, reduced graphene oxide, electrochemical synthesis

## Abstract

Films of a Ni/Al-layered double hydroxide intercalated with reduced graphene oxide were deposited, by means of a simple and rapid electrochemical synthesis, on Pt electrodes previously submitted to a special cleaning procedure. The aim of the research was to determine whether the better electrocatalytic properties of the Ni(III)/Ni(II) couple, due to the presence of the carbon nanomaterial, as compared to the Ni/Al-LDH alone, could allow glucose detection at physiological pHs, as normally LDHs work as redox mediators in basic solutions. Chronoamperometric experiments were carried out by applying a potential of 1.0 V vs. SCE to the electrode soaked in solutions buffered at pHs from 5.0 to 9.0 to which glucose was continuously added. The steady-state currents increased as the pH solution increased, but at pH = 7.0 the modified electrode exhibited a fast and rather sensitive response, which was linear up to 10.0 mM glucose, with a sensitivity of 0.56 A M^−1^ cm^−2^ and a limit of detection of 0.05 mM. Our results suggest the potential application of Ni/Al-LDH(ERGO) composite for the non-enzymatic detection of glucose or other oxidizable analytes under biological conditions.

## 1. Introduction

Looking for glucose determination in Scopus, it is possible to find more than 6500 papers in the last few years, and this fact is not surprising if we think about the importance that this molecule has in the medical field. The latest International Diabetes Federation (IDF) Atlas (2025) reports that 11.1% of the adult population (20–79 years) is living with diabetes, with over four in ten unaware that they have the condition. Accounting for around 90% of all diabetes, type 2 diabetes is the most common form of diabetes in the world. Diabetes was responsible for 3.4 million deaths in 2024, one every 9 s, and by 2050, IDF projections show that one in eight adults, approximately 853 million, will be living with diabetes, an increase of 46% [1]. Diabetes is a metabolic disease characterized by chronic hyperglycemia caused by multiple etiologies, generally related to impaired insulin secretion and insulin resistance [2].

The accurate measurement of glucose is important to monitor the condition of diabetic patients and to evaluate the effectiveness of treatment. Moreover, the management of all the related health complications needs a personal control of blood glucose daily, thus making glucose the most commonly tested analyte [3], which results in a huge expense for glucose biosensing point-of-care devices. Continuous Glucose Monitoring (CGM) devices represent technological advancements in diabetes management, as they offer a less intrusive method to measure glucose levels and, therefore, help improve the quality and style of life for type 2 diabetes patients, who are treated with insulin [4]. For all these reasons the development of new glucose sensing methodologies still represents a research target.

Electrochemical sensors, featuring high sensitivity and simple, low-cost equipment, have been employed to quantify many compounds of clinical interest [5]. Glucose sensors are broadly divided into two categories, namely, enzymatic and non-enzymatic. The advantages provided by non-enzymatic electrochemical glucose sensors have prompted the research for highly efficient, selective, and sensitive electrocatalysts [6].

Our research group has extensively studied electrochemical sensors based on chemically modified electrodes, exploiting their electrocatalytic properties, which are one of the distinguishing characteristics in electroanalytical chemistry. In particular, we were interested in layered double hydroxides (LDHs), containing Ni or Co, as electrode modifiers, due to their capability to act as redox mediators for the oxidation of electroactive analytes, coming from the redox behavior of the Ni(II)/Ni(III) or Co(III)/Co(IV) couples [7,8,9,10,11].

LDHs have the general formula [M(II)_1−x_M(III)_x_ (OH)_2_]^x+^ (A^n−^)_x/n_·yH_2_O, where M(II) and M(III) are metal cations that are bivalent and trivalent, respectively; x ranges from 0.22 to 0.33, and A^n−^ represents the interlayer anion, necessary to ensure the electroneutrality of the structure [12]. The main appeal of LDHs lies in the availability of a large range of compositions for both the metal cations in the hydroxide layers and the interlayer anions [13]. Therefore, LDHs represent very exciting materials due to their properties, such as lamellar structure, anion exchange capabilities for which they are also called anionic clays, a large surface-to-volume ratio (especially when synthesized as nanomaterials), a large number of adsorption-active sites, electrocatalytic behavior, chemical stability, and biocompatibility [8]. When transition metals (such as Co, Mn, and Ni) are present in the LDHs lamellae, the charge transport throughout the material takes place with a mechanism based on electron hopping and anion movements (from the solution into the clay during oxidation and vice versa during reduction). This mechanism is favored in alkaline media [8]. Even working in strongly basic conditions, however, LDHs are known to have low conductivities, thus showing poor electrochemical performance, especially for carbohydrate oxidation [9]. The main strategy to overcome this drawback is the combination of LDHs with carbon nanomaterials such as (graphene (G), reduced graphene oxide (RGO), and carbon nanotubes (CNS)) [7,14,15]. To enhance electrocatalytic properties and promote electronic and ionic transport in the electrodes modified with Ni-based LDHs, metal–organic framework (MOF) composites have also been proposed for non-enzymatic glucose sensing [16,17,18,19], or a MOF has been used as the sacrificial template [20] to develop electrochemical amperometric sensors with improved sensitivity and selectivity.

To the best of our knowledge, all the amperometric sensors exploiting Ni-based LDHs work in alkaline environments, usually 0.1 M NaOH, and this has limited the use of non-enzymatic glucose sensing systems in real-life measurements. In our opinion, detecting glucose at near-biological conditions, such as in an aqueous buffer at physiological pH, would expand the field of application of any kind of electrochemical sensor for biomolecules.

In 2021, our group electrosynthesized a composite based on Ni/Al-LDH and reduced graphene oxide by applying a cathodic potential scan, which allowed us to rapidly obtain, in a single step, both the production of the OH^−^ necessary for the precipitation of the LDH on Grafoil sheets and the electrochemical reduction of graphene oxide to obtain electrochemically reduced graphene oxide (ERGO) [21]. The resulting thin films were fully characterized from electrical and electrochemical points of view, and their performance was compared with thin films of the Ni/Al-LDH as such. Our results confirmed that the intercalation of ERGO among the layers of the LDH increased significantly both the conductivity of the composite material and the electrocatalytic properties of the couple Ni(III)/Ni(II), as it shifted the redox process to a less anodic potential [22]. In addition to improving the charge transport inside the composite, the ERGO layers also improve the stability of the clays, acting as a steric hindrance that slows down the reorganization processes occurring during the operation of the LDH-based electrochemical devices, as already demonstrated [23].

The aim of the present research was to test if the composite Ni/Al-LDH(ERGO) could be employed to modify electrode surfaces for the development of sensors capable of detecting and quantifying glucose at physiological pH.

## 2. Materials and Methods

### 2.1. Reagents

Boric, acetic, hydrochloric, and sulphuric acids and sodium hydroxide were purchased from Merck. Nickel (II) nitrate hexahydrate (Milan, Italy), Al(III) nitrate nonahydrate, potassium nitrate, potassium mono- and di-hydrogen phosphate, aqueous GO solution (4 mg mL^−1^), and glucose were purchased from Sigma-Aldrich (Merck Spa), Milan, Italy. All the reagents were analytical reagent grade and used as received.

The following buffer solutions (analytical concentration: 0.1 M) were prepared to investigate the pH effect on the performance of the electrodes: acetate (for pHs 5.0 and 6.0), phosphate (for pHs 7.0, 8.0, and 11.0), and borate (for pHs 9.0 and 10.0), starting from the proper acid or salt and adding NaOH or HCl to obtain the desired pH. All solutions were prepared with doubly distilled (DD) water.

### 2.2. Instrumentation

The electrochemical experiments were performed in a single-compartment, three-electrode cell. Electrode potentials were measured with respect to the aqueous saturated calomel electrode (SCE). A Pt wire was used as the counter electrode, and a Pt disk (2 mm diameter) modified with Ni/Al-LDH or Ni/Al-LDH(ERGO) was used as the working electrode. All the electrochemical experiments were recorded using a CHInstruments Mod. 660C, Bee Cave, TX, USA, controlled by a personal computer via CHInstruments sofware, version 17.2. For the ultrasonic treatment of the GO suspension, a Bandelin Sonorex Super Sonicator (RK 103 H), Berlin, Germany, was employed. An Amel pH meter (mod. 338), Milano, Italy, was used to measure the pH of the buffers.

The morphology of the LDH films was investigated by field emission-scanning electron microscopy (FE-SEM) using a LEO 1530 ZEISS instrument, Jena, Germany, equipped with a Schottky emitter, operated at an acceleration voltage variable from 5 to 15 kV, and an Everharte–Thornley (JEOL Ltd., Tokyo, Japan) and in-lens detectors for secondary electron imaging.

### 2.3. Fabrication of the Modified Electrodes

In order to obtain thin films of the composite Ni/Al-LDH(ERGO) on the Pt disk, the cleaning procedure of its surface is crucial. First, the Pt disk was polished to a mirror-like surface by mechanical cleaning using wet sandpaper (4000 grit), and then it was thoroughly rinsed with DD water. Later, it was submitted to an electrochemical treatment consisting of 250 cycles between −0.25 and +1.30 V in 0.1 M H_2_SO_4_ at a scan rate of 1.0 V s^−1,^ and then it was kept under stirring in 1 M H_2_SO_4_ at a potential of −0.5 V for 300 s. Finally, the electrode was again cycled 250 times between −0.25 and +1.30 V in 0.1 M H_2_SO_4_ at a scan rate of 1.0 V s^−1^.

For the electrodeposition of the LDH as such, the Pt disk was immersed in a freshly prepared solution containing Ni(NO_3_)_2_ and Al(NO_3_)_3_ in a molar ratio of 3:1 with respect to the cations. The total concentration of metal ions was 0.03 M. The LDH deposition was carried out by applying four cyclic voltammetry (CV) segments between −0.1 and −1.2 V vs. SCE at a scan rate of 0.040 V s^−1^. For the electrodeposition of the composite, the aqueous GO suspension (4 mg/mL) was added to the nitrate salts solution to obtain a final concentration of 0.05 mg/mL. Before the addition, the GO suspension was submitted to ultrasonic treatment for 20 min at 40% maximum power of the equipment. After the electrosynthetic procedure, the modified electrodes were thoroughly washed with DD water to remove traces of the electrolytic solution, which could partially dissolve the film, being slightly acidic [22].

### 2.4. Electrochemical Characterization

Once modified with the LDH or the composite, the Pt electrodes were submitted to 10 CV cycles, between 0.5 and 1.1 V, in 0.1 M NaOH, at a scan rate of 0.020 V s^−1^ in order to activate the electrocatalytic Ni centers and, above all, to stabilize the electrochemical signal during the following experiments. The electrochemical behavior of the electrodes was studied in 0.1 M NaOH by CV in a suitable potential window at the scan rate of 0.050 V s^−1^. In each experiment, CV curves were continuously recorded until the cycles resulted in superimposition. In a similar way, the electrodes modified with Ni/Al-LDH (ERGO) were also characterized in the buffer solutions in the pH range 5–11, washing the electrodes with DD water after each experiment.

### 2.5. Evaluation of the Catalytic Activity Toward Glucose

The catalytic activity of the electrodes for the determination of glucose was investigated in the buffer solution at pH 7.0 by chronoamperometry, working at 1.00 V. Subsequent additions of a standard solution of glucose were performed so that to work in a concentration range between 0.1 and 10.0 mM. Chronoamperometric experiments were performed also in the buffer solutions at the other investigated pHs.

## 3. Results and Discussion

### 3.1. Morphology of LDH and Composite

**Figure 1:**
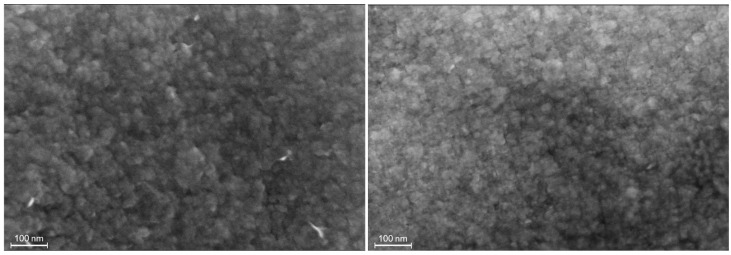
SEM images of Ni/Al-LDH (**left**) and Ni/Al-LDH(ERGO) (**right**) obtained at a magnification of 300,000×.

The morphology of the LDH films was investigated by means of FE-SEM (Figure 1). The first observation is that in both cases the Pt substrate is homogeneously covered, and no film detachment is observed. In both the syntheses, i.e., with and without GO, the film consists of a dense and homogeneous layer of nanostructured crystallites of around 20–40 nm.

The surface morphology appears unchanged in the presence of GO, although it is possible to observe that the crystallites are slightly smaller and more aggregated for the Ni/Al-LDH(ERGO) compared to the Ni/Al-LDH. In a previous work we developed the electrochemical procedure, which has been briefly described in Section 2.3, to synthesize in a single step the composite Ni/Al-LDH(ERGO) [21]. In that study we performed a structural characterization of the obtained nanostructured material, comparing it with the Ni/AL-LDH alone, by means of FT-IR, XRD, SEM, and TEM microscopies. The results confirmed the layered structure of the composite and the presence of ERGO intercalated inside the LDH layers.

### 3.2. Electrochemical Characterization

**Figure 2:**
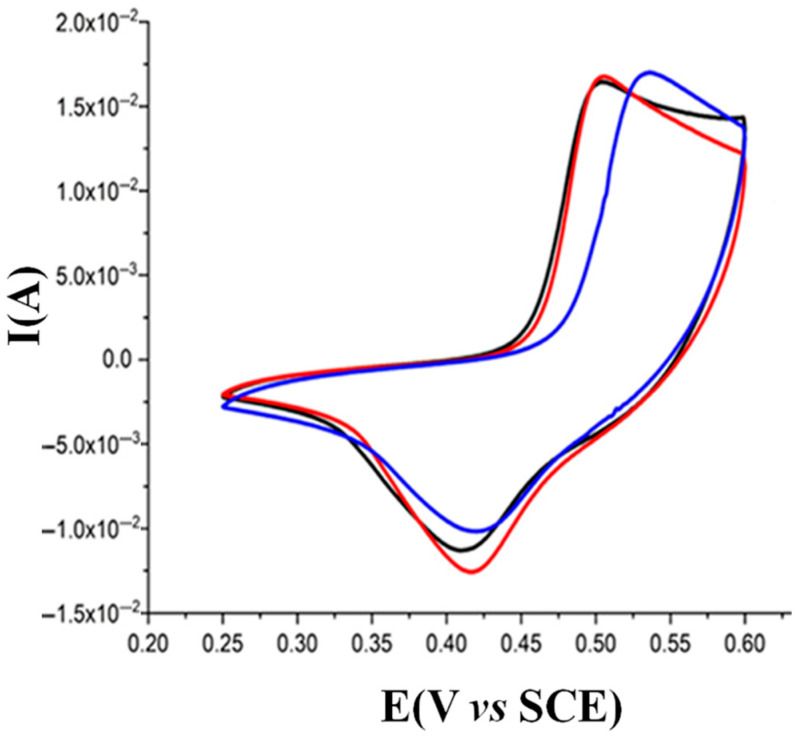
Characterization CVs of Pt electrodes modified with Ni/Al-LDH (blue trace) or Ni/Al-LDH(ERGO) with (red trace) and without (black trace) the activation treatment, recorded in 0.1 M NaOH.

The Pt electrodes modified with the LDH and the composite were electrochemically characterized by CV. The traces recorded in 0.1 M NaOH show a clearly visible Faradaic process, relevant to the Ni(III)/Ni(II) couple, which takes place at a formal potential of about 0.4 V and is characteristic of Ni/Al-LDH (Figure 2), according to the following reaction:

Ni(II)-LDH + OH^−^(aq) ⇄ Ni(III)(OH)-LDH + e^−^ (1)

As already stated in the Introduction, Reaction (1) is favored in alkaline solution, since OH^−^ ions are directly involved as reagents. Therefore, when increasing the NaOH concentration, the anodic peak potential moved to less positive values, and, in the meantime, the peak current increased (higher number of nickel sites involved in the electrochemical process). For Ni/Al-LDH(ERGO), the oxidation peak of Ni(II) occurs at a potential that is about 40 mV less anodic than for Ni/Al LDH. The same holds for the composite material after being submitted to the activation treatment consisting of 10 CV cycles, which also displays a major reversibility of the Ni(III)/Ni(II) redox process. Therefore, the presence of ERGO inside the Ni-based LDH facilitates the oxidation of Ni(II), thus improving the electrocatalytic properties of the composite. As already mentioned in the Introduction, the electrical characteristics of the composite have been investigated in a recent paper using electrochemical impedance spectroscopy, from which it was confirmed the role of ERGO in decreasing the impedance, especially in the low-frequency region, indicating an improved kinetics of the Ni centers redox reaction [22].

**Figure 3:**
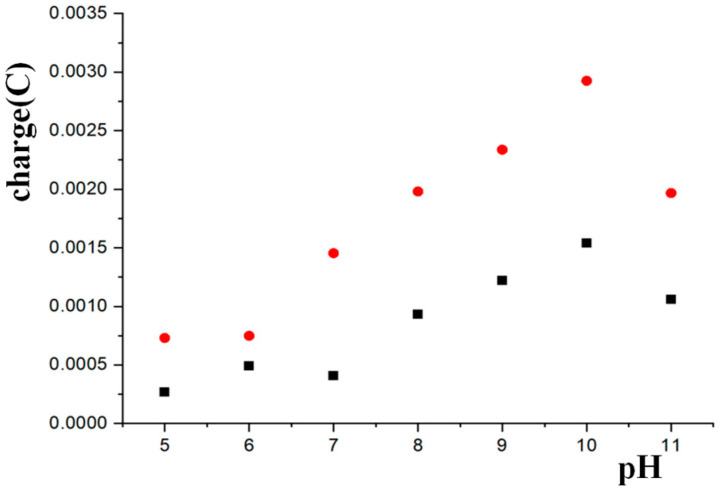
Charge passed during the characterization CVs for the oxidation of Ni(II) at the Ni/Al-LDH/Pt (black squares) and Ni/Al-LDH(ERGO)/Pt (red dots) electrodes soaked in the buffer solutions in the pH range from 5.0 to 11.0.

The electrodes Ni/Al-LDH/Pt and Ni/Al-LDH(ERGO)/Pt were characterized also in solutions buffered in the pH range 5–11. As the pH of the solutions decreases, the oxidation peak of Ni(II) moves to more anodic potentials, as already observed for the Co/Al LDHs in the pH ranges 10–12.8 [24]. The charge passed during the oxidation process involving Ni(II), calculated from the characterization CVs as a function of the electrolytic solution pH, is shown in Figure 3, from which it is clearly evident the positive linear dependence between the variables from pH = 5.0 to pH = 10.0. Moreover, as expected, at all the investigated pHs the charge is significantly higher when the modifier is the composite Ni/Al-LDH(ERGO) due to the more efficient electrochemical activity exhibited by the Ni(III) centers in the presence of the carbon nanomaterial inserted inside the LDH structure.

The drastic decrease of the charge when the pH is 11.0 cannot be considered anomalous, but it is instead related to the phosphate buffer, where the prevalent anion present in solution is monohydrogen phosphate, HPO_4_^2−^. In fact, one of the most outstanding properties of LDHs is their ability to act as anion exchangers. On the basis of the relative equilibrium constants, which increase as the anion charge increases, the following selectivity sequence for mono- and bivalent anions has been proposed [25]:

I. OH^−^ > F^−^ > Cl^−^ > Br^−^ > NO_3_^−^ > I^−^
II. CO_3_^2−^ > C_10_H_4_ N_2_O_8_S^2−^ > SO_4_^2−^
III. HPO_4_^2−^ > HAsO_4_^2−^ > CrO_4_^2−^ > SO_4_^2−^ > MoO_4_^2−^

For this reason, once HPO_4_^2−^ anions have been inserted among the positively charged LDH layers, they are so strongly retained by the material that Reaction (1) hardly can occur.

**Figure 4:**
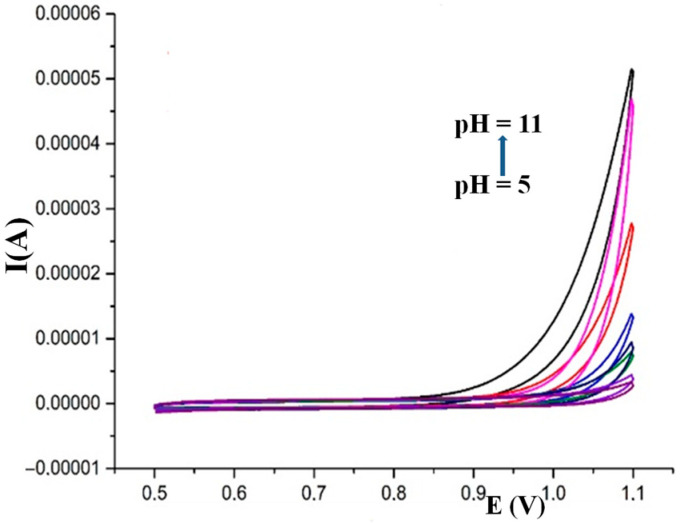
CVs were recorded at unmodified Pt electrodes in buffer solutions in the pH range from 5.0 to 11.0.

In order to verify that the electrochemical signals recorded during the characterization CVs do not depend on the presence of the Pt underlying the layers of the LDH or composite, CVs were also recorded for unmodified Pt electrodes, and the relevant traces are shown in Figure 4. It is possible to observe that the only current recorded at Pt electrodes is due to the oxygen evolution reaction, which occurs at high anodic potentials and is favored by the basic pH. In fact, at pH = 7.0 the reaction does not take place until the potential reaches values higher than 1.0 V.

### 3.3. Amperometric Detection of Glucose

**Figure 5:**
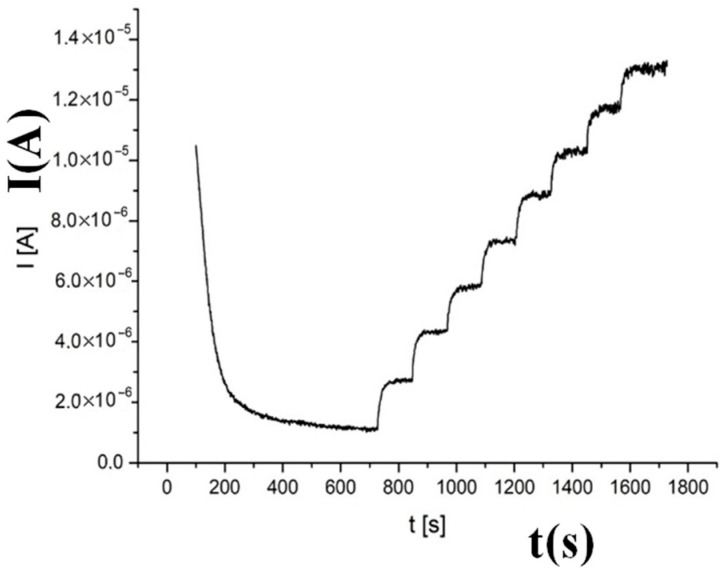
Amperometric response of a Ni/Al-LDH(ERGO)/Pt electrode, biased at 1.0 V vs. SCE, for continuous 1 mM additions of glucose to a solution buffered at pH = 7.0.

The current responses of a Ni/Al-LDH(ERGO)/Pt electrode to continuous additions of 1 mM glucose were recorded in phosphate solution at pH = 7.0, applying a potential of 1.0 V, and the results are presented in Figure 5. A stepwise increase in current density upon addition of glucose at increasing concentrations was observed until a stable current density was achieved; 90% of the steady-state current density was achieved in about 20 s. Therefore, the responsiveness of the composite material was excellent.

**Figure 6:**
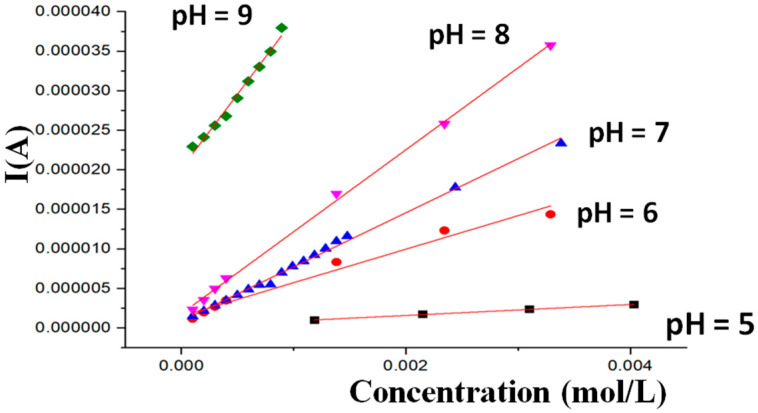
Calibration lines obtained for glucose sensing from the chronoamperometric experiments carried out with the Ni/Al-LDH(ERGO)/Pt electrode biased at 1.0 V vs. SCE in solutions buffered at pH from 5.0 to 9.0.

Chronoamperometry was also performed during continuous additions of 0.1 or 1 mM glucose to solutions buffered from pH 5.0 to 9.0. The electrode was responsive to glucose at all these pHs, with the current response gradually amplifying as the pH increased, as expected. The corresponding calibration lines are shown in Figure 6.

The sensitivity of the response is very low at pH = 5.0, but it becomes higher and higher as the pH increases, such that at pH 9.0, the chronoamperometric signals were recorded by making additions of 0.1 mM glucose solutions. Even if the results shown here are derived from a preliminary investigation carried out with the aim of demonstrating that electrodes modified with Ni/Al-LDH(ERGO) can be utilized for the detection of glucose at physiological pH, chronoamperometric experiments were repeated to determine the linearity range and the limit of detection (LOD) of the measurement. The electrode response was linear up to 10.0 mM glucose with a sensitivity equal to 0.56 A M^−1^ cm^−2^, calculated using the geometric area, and an LOD, based on the signal-to-noise ratio of 3 (S/N = 3) at 0.05 mM.

Taking into account that the normal concentration of glucose in blood for a healthy fasting individual is about 5.5 mM [26], it can be concluded that the performances of the Ni/Al-LDH(ERGO) electrode could be effectively exploited to develop a sensor for glucose in real blood samples, requiring only a simple centrifugation of the proper clinical tube and dilution of the plasma with phosphate buffer as pretreatment.

## 4. Conclusions

In summary, in the present study a fast and simple one-step electrochemical strategy was applied for the in situ growth of a Ni/Al-LDH(ERGO) composite on the surface of Pt substrates. The modified electrode was employed to electrocatalyze the oxidation of glucose at pHs from 5.0 to 11.0, thus demonstrating that it can be used for the non-enzymatic detection of glucose under biological conditions. That is made possible by the presence of the carbon-based nanomaterial, which improves the electrical conductivity of the LDH and facilitates the oxidation of Ni(II), resulting in an enhanced electrocatalytic effect.

Future studies will focus on improving the system with various strategies in order to minimize possible interferences and fouling of the modified electrode when employed for the determination of glucose or other oxidizable analytes in biological samples. It will also be possible to apply the same electrochemical modification procedure to screen-printed electrodes with the aim of developing disposable sensors for applications in glucose-sensing point-of-care devices.

## Data Availability

The original contributions presented in this study are included in the article. Further inquiries can be directed at the corresponding author.
